# Calreticulin Mutations in Myeloproliferative Neoplasms

**DOI:** 10.5041/RMMJ.10169

**Published:** 2014-10-29

**Authors:** Noa Lavi

**Affiliations:** Department of Hematology and Bone Marrow Transplantation, Rambam Health Care Campus, Haifa, Israel; and Bruce Rappaport Faculty of Medicine, Technion–Israel Institute of Technology, Haifa, Israel

**Keywords:** Calreticulin, essential thrombocythemia, myeloproliferative neoplasms, primary myelofibrosis

## Abstract

With the discovery of the *JAK2*V617F mutation in patients with Philadelphia chromosome-negative (Ph^−^) myeloproliferative neoplasms (MPNs) in 2005, major advances have been made in the diagnosis of MPNs, in understanding of their pathogenesis involving the JAK/STAT pathway, and finally in the development of novel therapies targeting this pathway. Nevertheless, it remains unknown which mutations exist in approximately one-third of patients with non-mutated *JAK2* or *MPL* essential thrombocythemia (ET) and primary myelofibrosis (PMF). At the end of 2013, two studies identified recurrent mutations in the gene encoding calreticulin (*CALR*) using whole-exome sequencing. These mutations were revealed in the majority of ET and PMF patients with non-mutated *JAK2* or *MPL* but not in polycythemia vera patients. Somatic 52-bp deletions (type 1 mutations) and recurrent 5-bp insertions (type 2 mutations) in exon 9 of the *CALR* gene (the last exon encoding the C-terminal amino acids of the protein calreticulin) were detected and found always to generate frameshift mutations. All detected mutant calreticulin proteins shared a novel amino acid sequence at the C-terminal. Mutations in *CALR* are acquired early in the clonal history of the disease, and they cause activation of JAK/STAT signaling. The *CALR* mutations are the second most frequent mutations in Ph^−^ MPN patients after the *JAK2*V617F mutation, and their detection has significantly improved the diagnostic approach for ET and PMF. The characteristics of the *CALR* mutations as well as their diagnostic, clinical, and pathogenesis implications are discussed in this review.

## INTRODUCTION

The classic Philadelphia chromosome-negative (Ph^−^) myeloproliferative neoplasms (MPNs) include polycythemia vera (PV), essential thrombocythemia (ET), and primary myelofibrosis (PMF). In 2005, with the discovery of the Janus kinase 2 (*JAK2*) V617F mutation, a major advance has been made in understanding the pathogenesis of increased signaling by the JAK/STAT pathway in MPNs.^1^–^4^ The *JAK2*V617F mutation is present in 95%, 50%, and 60% of PV, ET, and PMF patients, respectively.^5^ Two other mutations (*JAK2* exon 12 and mutation in the thrombopoietin receptor gene, myeloproliferative leukemia, *MPL*) directly affecting this pathway were then described. The *JAK2* exon 12 mutation is present in 2% of PV patients; the *MPL* mutation is present in 5% and 10% of patients with non-mutated *JAK2* ET and PMF patients, respectively.^6^ While somatic mutations observed in other genes, such as *TET2*, *ASXL1*, *DNMT3A*, and *EZH2*, are found in MPNs, they are revealed in patients both with and without *JAK2* and *MPL* mutations and are not specific to these disorders.

Until recently, in approximately one-third of the patients with non-mutated *JAK2* or *MPL* ET and PMF, the driver mutation had not been recognized.

In 2013, studies by Klampfl et al.^7^ and Nangalia et al.^8^ identified recurrent mutations in the gene encoding calreticulin (*CALR*) in the majority of patients with non-mutated *JAK2* or *MPL*, and provided evidence for their role in the development of MPNs. Results of further studies, looking at the role of *CALR* mutations in the pathogenesis of MPNs and the clinical relevance of these mutations, have been reported. An overview of our knowledge regarding these *CALR* mutations in MPNs is given herein.

## THE DISCOVERY OF *CALR* MUTATIONS

Klampfl et al.^7^ performed exome sequencing from peripheral blood granulocyte DNA (tumor samples) and matched CD3+ T-lymphocyte DNA (control samples) in six patients with *JAK2* and *MPL*-negative PMF. Two to 12 somatic mutations per patient were found, and the only recurrently affected gene was *CALR*, a gene located on chromosome 19p13.2 and containing nine exons, encoding calreticulin. Two patients had somatic deletions, and four patients had a recurrent 5-bp insertion in exon 9 of *CALR* (the latter exon encoding the C-terminal amino acids of the protein).

A total of 896 patients with MPNs were then screened by polymerase chain reaction for insertion and deletion mutations in *CALR* exon 9. Mutations in *CALR* were not found in PV patients. Twenty-five percent and 35% of ET and PMF patients, respectively, had mutations in *CALR*. All patients with mutated *CALR* had non-mutated *JAK2* and *MPL* (*CALR* mutations are mutually exclusive with mutations in both *JAK2* and *MPL*).

Less than 10% of patients with ET or PMF were negative to *JAK2*, *MPL*, and *CALR* mutations. Several studies analyzing *CALR* mutations in MPN have been published since then, reporting triple-negative (negative to *JAK2*, *MPL*, and *CALR* mutations) ET prevalence of 10%–23%.^9^–^11^

Nangalia et al.^8^ performed exome sequencing on samples from 151 patients with myeloproliferative neoplasms. Somatic mutations in *CALR* were identified in 26 of 31 patients with ET or PMF with non-mutated *JAK2* or *MPL*.

*JAK2*V617F and *CALR* exon 9 mutations were deemed to be mutually exclusive. However, three cases (two ET patients and one patient with PMF) of double mutations (*JAK2*V617F and *CALR* mutation) have been reported.^12^–^14^ The true frequency and the pathogenic and clinical meaning of double mutation are not known yet.

Patients with other myeloid disorders (acute myeloid leukemia, chronic myeloid leukemia, myelodysplastic syndrome (MDS), chronic myelomonocytic leukemia, refractory anemia with ring sideroblasts associated with marked thrombocytosis (RARS-T)) were screened for mutations in *CALR* exon 9. Three patients with RARS-T had mutations in *CALR*.^7^ Mutations in *CALR* were identified in 8% of patients with MDS.^8^ The mutation was found in 1 of 524 healthy volunteers.^7^

Thirty-six types of somatic mutations in *CALR* (insertions and deletions) that caused a frameshift to an alternative reading frame were detected. Mutations of type 1 (52-bp deletion) and mutations of type 2 (5-bp insertion) accounted for 53.0% and 31.7% of all the cases with mutated *CALR*, respectively. Type 1 mutations were significantly more frequent in PMF than in ET. Other mutation types were observed at much lower frequencies.

## CALRETICULIN PROTEIN

Calreticulin is a protein with multiple reported functions. Within the endoplasmic reticulum, the protein ensures appropriate folding of newly synthesized glycoproteins and modulates calcium homeostasis.^15^,^16^ Calreticulin is also found in intracellular, cell-surface, and extracellular compartments, where it has been implicated in many biologic processes, including proliferation, apoptosis, and immunogenic cell death.^17^–^20^

Calreticulin has three main structural and functional domains: an N-terminal lectin-binding domain, a proline-rich P domain, and a C-terminal acidic domain that contains multiple calcium-binding sites. Calreticulin contains the endoplasmic reticulum–retention motif (KDEL motif) at the C-terminal end. The KDEL motif is present on some endoplasmic reticulum proteins and enables retrieval of these proteins from the Golgi apparatus back to the endoplasmic reticulum.

## MUTANT CALRETICULIN PROTEIN WITH AN ALTERED C-TERMINAL

All detected mutant *CALR* proteins share a novel amino acid sequence at the C-terminal. The non-mutant *CALR* C-terminal is largely negatively charged, whereas the mutant *CALR* C-terminal contains a number of positively charged amino acids. Type 1 mutations eliminate almost all negatively charged amino acids, whereas the type 2 mutations retain approximately half the negatively charged amino acids. Since the negatively charged C-terminal domain of calreticulin is the low-affinity, high-capacity, Ca^2+^-binding domain, the Ca^2+^-binding function of the mutant protein may be impaired. Additionally, the KDEL motif at the C-terminal end is lost in all mutant variants. Consequently, mutant calreticulin may have an altered subcellular localization.

To investigate whether mutations in *CALR* are acquired early or late in the clonal history of a patient, hematopoietic progenitor colonies obtained from MPN patients were analyzed, and it was found that the mutations in *CALR* were acquired early in the major clones.^7^,^8^

## THE EFFECT OF *CALR* MUTATIONS ON THE PATHOGENESIS OF MPN

Non-mutated and type 1 mutated *CALR* were transfected into an interleukin-3-dependent murine cell line. Cells expressing the type 1 *CALR* mutation showed growth that was independent of interleukin-3 and also showed hypersensitivity to interleukin-3. Cells expressing the non-mutated *CALR* or the type 1 mutation of *CALR* demonstrated similar sensitivity to *JAK2* kinase inhibitor, suggesting that the interleukin-3-independent growth of the mutated *CALR* cells depends on *JAK2* or a JAK family kinase. To confirm this hypothesis, phosphorylation of STAT5 was examined in the presence and absence of interleukin-3 in the control and *CALR-*transfected cell lines. Increased phosphorylation of STAT5 was detected in the absence of interleukin-3 and in low concentration of interleukin-3 in the type 1 mutation of *CALR* cells but not in the non-mutated *CALR* cells. These findings support the assumption of the activation of JAK/STAT signaling in type 1 *CALR* mutations.

## FAMILIAL MPNS

Although most MPN cases are sporadic, familial MPNs (at least two members have a MPN) are well described. Familial MPN members may have somatically acquired mutations. It is believed that patients with familial MPN inherit the “predisposition” to develop MPN somatic mutations. A study among 21 patients with familial MPNs found two members with the *CALR* mutation, demonstrating that these mutations may also occur in familial cases.^21^

## CLINICAL RELEVANCE OF *CALR* MUTATIONS IN MPNS

Mutant *CALR* is associated with younger age and male sex in ET patients^10^,^22^ and with younger age in PMF patients.^13^

### Blood Counts

Among patients with ET, those with a *CALR* mutation had a lower hemoglobin level, a lower white blood cell (WBC) count, and a higher platelet count at diagnosis than patients with mutated *JAK2*.^7^–^10^,^22^ Patients with *JAK2*V617F had a lower serum erythropoietin than those with *CALR* mutation.

Among patients with PMF, those with a *CALR* mutation had a lower WBC and a higher platelet count at diagnosis than patients with mutated *JAK2*.^7^ In a univariate analysis performed in 254 patients with PMF,^13^ *CALR* mutations were associated with a higher platelet count (*P*<0.0001). Patients with *CALR* mutations were also less likely to be anemic, require transfusions, or display leukocytosis.

These findings together with the detection of *CALR* mutations also in patients with RARS-T support a causal relationship between *CALR* mutations and excessive platelet production.

### Thrombosis Risk

Among patients with ET, those with a *CALR* mutation had a lower risk of thrombosis than did those with the *JAK2* mutation.^7^,^9^,^10^ In a cohort of 144 patients with splanchnic vein thrombosis (SVT), the incidence of *JAK2*V617F mutation was 18.8%; *CALR* exon 9 mutations were not detected in any of the 144 SVT patients. This finding supports the lower risk for thrombosis in patients with *CALR* mutations compared to patients with *JAK2* mutations.^23^

### Polycythemic Transformation

While no polycythemic transformation was observed in *CALR*-mutated patients, the cumulative risk was 29% at 15 years in those with *JAK2*-mutated ET.^9^

### Transformation to Myelofibrosis

There are conflicting results regarding the incidence of transformation to myelofibrosis (MF) according to the somatic mutational status. In one study, patients with *CALR* mutations had a significantly higher incidence of transformation from ET to MF than those with *JAK2* mutations.^8^ In other studies,^9^,^22^ there was not a significant difference in myelofibrotic transformation between these two groups.

### Overall Survival

In the study by Klampfl et al., a multivariate analysis demonstrated that MPN patients with *JAK2* and *MPL* mutations had a higher risk of death than ET and PMF patients with *CALR* mutations.^7^ In the study by Nangalia et al.^8^ no apparent survival difference was found between the two ET mutational groups. In a cohort of 576 ET patients,^10^ the *CALR* mutation did not influence the risk of death. The impact of *CALR* mutations on long-term survival in ET was also examined in 299 patients whose diagnosis pre-dated 2006.^22^ Survival was longest for triple-negative and shortest for *MPL*-mutated patients. Median survival was 19 years for *JAK2* and 20 years for *CALR*-mutated patients (*P*=0.32). This study is uniquely characterized by its very long follow-up period, provides accurate estimates of long-term survival in ET, and complements current information on mutation-specific phenotype and prognosis.

In PMF, *CALR* mutations had a favorable impact on survival that was independent of both Dynamic International Prognostic Scoring System (DIPSS)-plus risk and *ASXL1* mutation status.^13^ Triple-negative patients displayed inferior leukemia-free survival. These findings identify “*CALR*(−)/*ASXL1*(+)” and “triple-negative” as high-risk molecular signatures in PMF.

In a subsequent study,^24^ 570 PMF patients were recruited for derivation (*n*=277) and validation (*n*= 293) of a molecular prognostic model based on *CALR* and *ASXL1* mutations. Survival was the longest in *CALR*(+)/*ASXL1*(−) and shortest in *CALR*(−)/*ASXL1*(+) patients. The *CALR/ASXL1* mutation-based prognostic model was DIPSS-plus independent and effective in identifying low–intermediate-1-risk patients and high–intermediate-2-risk patients with a shorter or longer survival.

Comparison of type 1 versus type 2 *CALR* mutations in PMF showed the latter to be associated with higher-risk DIPSS-plus scores, *EZH2* mutations, marked leukocytosis, and increased peripheral blast percentage. Survival was significantly longer in patients with type 1 *CALR* mutations compared with both *JAK2*- and type 2 *CALR* mutations. This study suggests that the favorable prognostic impact of *CALR* mutations on PMF might be restricted to patients with type 1 *CALR* mutations.^25^

## CURRENT DIAGNOSTIC APPROACH IN ET AND PMF

The discovery of the *JAK2*V617F mutation resulted after just a few years in an exceptional amount of new information in the field of MPN. One important consequence of the new findings was the modification of the World Health Organization (WHO) classification and diagnostic algorithms for these diseases. *JAK2*-positive MPN patients share some features, but currently available data do not firmly support any different management due to the presence or absence of the *JAK2* mutation, and the WHO classification remains the diagnostic tool used in clinical practice.^26^

**Figure 1.**
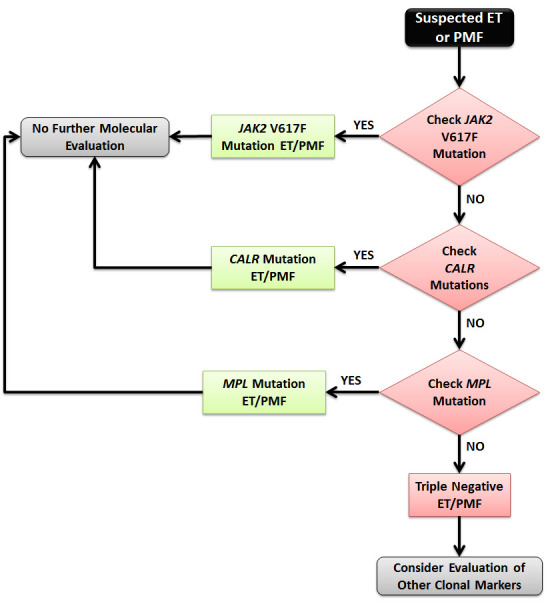
**Molecular Work-up Algorithm for the Diagnosis of ET or PMF.**

The discovery of *CALR* mutations is another milestone in our understanding of the pathogenesis of MPNs. The assessment of *CALR* mutations significantly improves the diagnostic approach for ET and PMF. In the suspicion of ET or PMF, initial mutation screening should start with the assessment of *JAK2*V617F mutation and then proceed with *CALR* mutation screening only in patients who are *JAK2*V617F-negative. Patients who are negative for the *CALR* mutation should then be screened for the *MPL* mutation. Triple-negative are those who are negative for the three mutations (Figure 1).^27^ Screening for *CALR* mutation should now be included in the diagnostic work-up of MPN and formally incorporated in future revisions of the WHO classification system.^28^ Similar to the *JAK2*V617F mutation, attempts to create a molecular classification of MPN according to *JAK2*/*MPL*/*CALR* mutations are still premature but may be the future of MPN.^29^

## TREATMENT IMPLICATIONS

Essential thrombocythemia patients with *CALR* mutations are at a lower risk of thrombotic complications compared to those with *JAK2* mutations; however, recommendations regarding modifications of current treatment based on mutation status have not been developed yet.

In two patients with essential thrombocythemia and *CALR* mutations, therapy with interferon alpha resulted in a sustained hematologic complete response, maintained after the discontinuation of therapy, and in reduction of the mutant allele burden, suggesting that *CALR*-mutated cells can be targeted by interferon alpha, with achievement of durable responses.^30^

Two clinical trials on the use of ruxolitinib for the treatment of PMF have shown that this *JAK1* and *JAK2* inhibitor is effective in most patients regardless of whether they have the *JAK2*V617F mutation.^31^,^32^ The fact that the vast majority of patients with non-mutated *JAK2* have a *CALR* mutation implies that ruxolitinib is also effective in patients with *CALR* mutations.^33^

According to the molecular prognostic model based on *CALR* and *ASXL1* mutations, stem cell transplant should be considered not only for DIPSS-plus high-risk myelofibrosis but also for any-risk disease with *CALR*(−)/*ASXL1*(+) mutational status.^34^ More studies are needed to validate the association of “*CALR*(−)/*ASXL1*(+)” and “triple-negative” mutation profiles with poor prognosis in PMF. I believe that mutational profile will be integrated in a future risk stratification score for myelofibrosis, which will help selecting high-risk patients for allogeneic transplantation.

## SUMMARY

Tables 1 and 2 summarize various clinical and therapeutic particularities of ET and PMF, according to *JAK2* or *CALR* molecular subtypes.

**Table 1.** The Various Clinical and Therapeutic Particularities of ET According to *JAK2* or *CALR* Molecular Subtypes.

		***JAK2*V617F-mutated ET**	***CALR*-mutated ET**
Age, sex		Older	Younger, M>F
Blood counts	Hemoglobin	Higher	Lower
	Platelet	Lower	Higher
	White blood count	Higher	Lower
Serum erythropoietin level		Lower	Higher
Thrombotic risk		Higher	Lower
Polycythemic risk		Cumulative risk 29% at 15 years	No transformation to PV
MF transformation risk			Conflicting results
Overall survival (OS)		Long-term OS similar to that in *CALR*-mutated ET	Long-term OS similar to that in *JAK2*V617F-mutated ET
Treatment implications			ET patients with *CALR* mutations are at a lower risk of thrombotic complications compared to those with *JAK2* mutations; however, recommendations regarding modifications of current treatment based on mutation status have not been developed yet

**Table 2.** The Various Clinical and Therapeutic Particularities of PMF According to *JAK2* or *CALR* Molecular Subtypes.

		***JAK2*V617F-mutated ET**	***CALR*-mutated ET**
Age		Older	Younger
Blood counts	Hemoglobin	Higher	Lower
	Platelet	Lower	Higher
	White blood count	Higher	Lower
Overall survival			Longer, may be restricted only to patients with type 1 *CALR* mutations
Treatment implications		Stem cell transplant should be considered not only for DIPSS-plus high-risk myelofibrosis but also for any-risk disease with *CALR*(−)/*ASXL1*(+) mutational status	

## FUTURE PERSPECTIVES

Additional studies are needed to understand the functional relevance of *CALR* mutations in the pathogenesis of MPNs and to determine the influence of these mutations on disease phenotype.

The presence of the peptide sequence derived from an alternative reading frame at the C-terminal domain of mutated *CALR* offers an opportunity for immunologic targeting because it represents a cancer-specific epitope.

## Abbreviations:

CALR: calreticulin;
DIPSS: Dynamic International Prognostic Scoring System;
ET: essential thrombocythemia;
JAK2: Janus kinase 2;
MDS: myelodysplastic syndrome;
MPL: myeloproliferative leukemia;
MPNs: myeloproliferative neoplasms;
Ph: Philadelphia chromosome;
PMF: primary myelofibrosis;
PV: polycythemia vera;
SVT: splanchnic vein thrombosis;
WBC: white blood cell.
